# The impact of workplace psychological violence on clinical nurses' turnover intention: the mediating role of perceived stress

**DOI:** 10.3389/fpubh.2025.1672644

**Published:** 2025-11-17

**Authors:** Lin Tan, Shouqi Zheng, Xiaoli Zhong, Ying Han, Lin Xia, Yuting Fan, Lin He

**Affiliations:** 1 Department of Nursing, Deyang People's Hospital, Deyang, China; 2 School of Nursing, North Sichuan Medical College, Nanchong, China

**Keywords:** nurses, turnover intention, workplace psychological violence, perceived stress, mediating effect

## Abstract

**Background:**

The global shortage of nurses has become a significant health emergency, and nurses' turnover intention is a key influencing factor, serving as an important predictor of actual turnover rates. However, studies integrating workplace psychological violence, perceived stress, and turnover intention in the same mediation model are still limited, so there is an urgent need to explore in depth the mediating effect of perceived stress between workplace psychological violence and turnover intention.

**Objective:**

To examine the mediating effect of nurses' perceived stress on the relationship between workplace psychological violence and turnover intention among nurses in tertiary general hospitals in Southwest China.

**Methods:**

Between October 2024 and March 2025, 798 nurses from nine tertiary general hospitals in southwest China were selected by a convenience sampling method for the study. A cross-sectional survey was conducted using the General Information Questionnaire, Turnover Intention Scale, Psychological Violence in the Workplace Scale, and Perceived Stress Scale. The mediating effect was examined using SPSS and PROCESS Model 4.

**Results:**

Turnover intention was positively correlated with Workplace Psychological Violence (*r* = 0.364, *P* < 0.001) and with Perceived Stress (*r* = 0.423, *P* < 0.001), and Workplace Psychological Violence was positively correlated with Perceived Stress (*r* = 0.486, *P* < 0.001). Perceived stress showed a partial mediating effect in the influence of psychological violence in the workplace on turnover intention, with a mediating effect value of 0.129, and the mediating effect accounted for 36.96% of the total effect.

**Conclusion:**

Perceived stress demonstrates a mediating effect between workplace psychological violence and turnover intention among nurses. Nursing managers can reduce perceived stress among nurses by decreasing workplace psychological violence, thus reducing nurses' turnover intention, improving nurses' job satisfaction and mental health, and promoting the stability and sustainable development of the nursing team.

## 1 Introduction

A new report from the International Council of Nurses (ICN) states that the global shortage of nurses should be recognized as a global health emergency (1). The global turnover rate of nurses ranged from 8 to 36.6%, with an overall turnover rate of 16%, with a significantly higher turnover rate in Asia (19%) than in North America (15%), which is mainly attributed to the differences in economic and cultural systems, different hospital management models, and varying degrees of nurse shortages among other regions (2). Another study yielded similar results, showing that Asian countries had the highest nursing turnover rate (20%), followed by North American countries (15%) and European countries (7%), further confirming the significant differences in nurse turnover rates between regions (3). Nurses' turnover intention is a key factor influencing the global nursing shortage and is an essential predictor of the actual turnover rate (4, 5). Turnover intention refers to an employee's behavioral intention or attitude toward leaving their organization or unit, a state of mind or tendency that precedes actual departure (6). Nurses leaving their jobs can negatively impact nurse staffing, nurse outcomes, and patient outcomes, ultimately decreasing the quality of care and harming patients, while also generating unnecessary healthcare expenditures (7, 8). It has also been shown that nurse staffing is associated with patient mortality and patient outcomes (9). Nurses' turnover intention is negatively correlated with nursing outcomes, and reducing nurses' turnover intention can lead to retention of nurses, which can further benefit patients in terms of various nursing outcomes (10).

Studies have shown that the higher the level of psychological violence experienced by nurses in the workplace, the higher the turnover intention (11, 12). Workplace psychological violence among nurses refers to any act of psychological violence that occurs in the workplace without physical contact, such as workplace bullying, verbal aggression, intimidation and threat (13). Chronic exposure to workplace psychological violence can lead to decreased job satisfaction, emotional exhaustion, and increased psychological distress among clinical nurses (14). Increased turnover intention is associated with experiencing adverse workplace events, high job stress, and high work-family conflict, and low turnover intention is related to good person-organization and person-group fit (15). Failure to address workplace psychological violence may exacerbate existing challenges in healthcare.

Stress is an essential factor that influences turnover intention (16). Stress, as one of the leading causes of physical and mental problems in healthcare workers and reduced productivity in healthcare organizations, negatively affects the quality of life and care for nurses, and the health harm it causes may jeopardize patient safety (17, 18). Perceived stress refers to the tension that arises from an individual's subjective cognitive appraisal of external environmental threats or stressful events (19). Yuan et al. have found that female healthcare workers generally have higher levels of perceived stress, which is a direct and positive predictor of their turnover intention (20). At the same time, there was also a significant positive correlation between perceived stress and violence (21).

Existing research indicates that nurses' turnover intentions are associated with psychological violence in the workplace and perceived stress, but the underlying mechanisms linking these three factors remain incompletely understood. The Job Demands-Resources Model (JD-R) (22) provides a crucial theoretical framework for understanding this complex relationship. This model describes job characteristics through two dimensions: job demands and job resources. Job demands refer to aspects of work that require sustained physical and mental effort, consuming individual energy. Job resources, conversely, are aspects of work that facilitate achieving job goals, reduce the costs of demands, or promote personal development (23). The core of the JD-R model lies in two process mechanisms: when job demands exceed personal resources, it activates a health-damaging process, leading to stress experiences and adverse work outcomes; conversely, sufficient job resources activate a motivational process, promoting positive work attitudes and behaviors (24). Based on this theoretical logic, when individuals face high job demands without adequate resources, they experience psychological strain and stress through a health-damaging process, affecting their work attitudes and behavioral intentions. There is still limited research that integrates workplace psychological violence, perceived stress, and turnover intention in the same mediational model. Therefore, this study proposes the hypothesis that perceived stress may mediate workplace psychological violence and turnover intention. Exploring the mediating mechanism of perceived stress between psychological violence and turnover intention not only contributes to a deeper understanding of the factors influencing nurses' turnover intention but also provides a reference for developing targeted interventions, which can alleviate the problem of clinical nurse shortage to some extent and improve the quality of healthcare services.

## 2 Objects and methods

### 2.1 Research design

A cross-sectional survey of clinical nurses in nine tertiary general hospitals in southwest China was conducted using convenience sampling from October 2024 to March 2025.

### 2.2 Participants

Inclusion criteria: Registered nurses employed in general hospitals in Southwest China; Engaged in clinical nursing for ≥6 months. Exclusion criteria; Nurses in continuing education, internship, rotation, or participating in standardized residency training; Those unable to participate due to leave of absence or temporary assignment elsewhere.

### 2.3 Sample size calculation

The required sample size was calculated using the formula for proportion estimation: n = u^2^ α/2π(1-π)/ δ^2^ where u represents the critical value from the standard normal distribution. At a 95% confidence level (two-sided), u = 1.96; π represents the proportion set at 42.42% based on the reported turnover intention rate among Chinese nurses (25); and δ represents the allowable error, set at 5%. Based on this formula, the calculated sample size was 376 participants. Accounting for a 20% invalid response rate, a minimum sample size of 470 participants was determined appropriate for this study.

### 2.4 Survey tools

#### 2.4.1 General demographic questionnaire

A self-developed demographic questionnaire was used for data collection. The instrument was designed by the research team through a literature review and panel discussions to identify key variables relevant to the study population. The questionnaire covered demographic and work-related characteristics, including gender, age, marital status, having children or not, highest educational attainment, professional title, job position, type of employment, department, length of service in nursing, average daily working hours, monthly income, number of night shifts per month, and recent major life events.

#### 2.4.2 Turnover intention scale

This scale was developed by Michaels and Spector in 1982 (26) and adapted into Chinese by scholars, including Li and Li (27), for assessing turnover intention. It is widely used among nursing populations (28, 29). The scale comprises three dimensions with six items: possibility of quitting (two items), motivation to find other jobs (two items), and possibility of obtaining other jobs (two items). Each item employs a 4-point Likert scale ranging from “never” to “often,” scored 1–4 points respectively. Total scores range from 6 to 24 points, with higher scores indicating stronger turnover intention. The scale demonstrates good reliability and validity, with an overall Cronbach's α coefficient of 0.773. In this study, the Cronbach's α coefficient for the scale was 0.831.

#### 2.4.3 Workplace psychologically violent behaviors instrument

This scale was developed by Turkish scholars Dilek and Aytolan (30) in 2008 and subsequently translated and revised by Xu et al. (31). It is now widely used to measure the extent of psychological violence experienced by clinical nurses in the workplace (32, 33). The scale comprises 32 items across four dimensions: individual isolation work (10 items), attack on professional status (nine items), attack on personality (seven items), direct negative behaviors (six items). Using the Likert 6-point scoring method, a score of 0–5 from “never happened” to “always” is assigned. The total score is the sum of all 32 items, ranging from 0 to 160. Higher scores represent a higher frequency of workplace psychological violence. The overall Cronbach's α for the scale is 0.964, and the content validity index is 0.875, demonstrating good reliability and validity. In this study, the Cronbach's α coefficient was 0.975.

#### 2.4.4 Perceived stress scale

This scale was developed by Cohen et al. (19) and adapted and revised for the Chinese context by Yang et al. (34) to measure nurses' perceived stress levels (35, 36). It comprises two dimensions with 14 items: sense of tension (seven items) and sense of losing control (seven items). The scale employs a 5-point Likert scale with response options ranging from “Never” to “Always.” Items are scored on a scale of 1–5 points, with some items reverse-scored The total score is the sum of all 14 items, ranging from 14 to 60 points, where a higher score indicates a greater level of perceived stress among nurses. The scale demonstrates good reliability and validity, with an overall Cronbach's α of 0.78 and item factor loadings ranging from 0.50 to 0.78. In this study, the Cronbach's α coefficient for the scale was 0.812.

### 2.5 Ethical considerations

The study was conducted following the Declaration of Helsinki and approved by the Ethics Committee of Deyang People's Hospital (Ethics No. 2024-08-025-K04). This study adheres to the principle of voluntary participation and implements informed consent through the Wenjuanxing online platform: an informed consent page is displayed before the questionnaire begins, detailing the research objectives, content, participant rights, data usage methods, and researcher contact information. Participants must actively check the consent box before proceeding to the formal questionnaire. Participants can refuse or withdraw from the study at any time without incurring any loss of benefits. This study employs an anonymous completion method and does not collect personally identifiable information. All raw data will be stored confidentially and accessible only to principal investigators.

### 2.6 Data collection

The researcher first contacted and obtained approval and support from the nursing department director of each hospital, and then screened for suitable study participants in strict accordance with the inclusion and exclusion criteria. Online data collection was conducted using the Wenjuanxing online survey platform, with each IP address restricted to one submission. All questions were designated as mandatory. To ensure data quality, two researchers independently reviewed completed questionnaires after collection, excluding invalid responses with abnormal completion times, patterns of consistent answers, or logical inconsistencies. A total of 880 questionnaires were distributed, with 798 valid responses ultimately collected, achieving a valid response rate of 90.68%.

### 2.7 Data analysis

Statistical analyses were conducted using SPSS 26.0 software. Normality tests were performed on all continuous variables. The count data were described using frequencies and percentages. In contrast, continuous data were described using means and standard deviations to analyze participants' sociodemographic characteristics and scores on turnover intention, workplace psychological violence, and perceived stress. Independent *t*-tests and analysis of variance (ANOVA) were used to compare differences in turnover intention across participants' sociodemographic characteristics. Correlations between nurses' turnover intention, workplace psychological violence, and perceived stress were examined using Pearson correlation analysis. Mediation effects were analyzed using Model 4 of the PROCESS macro with 95% confidence intervals, employing the bootstrap method with 5,000 resamples at a significance level of α = 0.05.

## 3 Results

### 3.1 Common method bias test

Harman's single-factor test was conducted on all questionnaire items to assess common method bias. The results showed that eight factors had eigenvalues greater than 1.0, and the first unrotated factor accounted for 39.508% of the total variance, which did not exceed the critical threshold of 40%. This indicates that the study did not suffer from serious standard method bias.

### 3.2 Demographic characteristics of participants

Table 1 shows the sociodemographic characteristics of the 798 study participants in detail. Female nurses comprised 767 participants (96.1%), reflecting the gender distribution of the nursing profession. Regarding age structure, the 31–40 age group represented the most significant proportion with 322 nurses (40.4%). For family status, 71.8% were married, and 548 (68.7%) had children, indicating that most participants had established families. Regarding educational and professional background, bachelor's degree holders dominated the sample with 584 participants (73.2%). Nurse supervisors represented the highest proportion (40.9%), while clinical nurses comprised the majority of positions (78.2%). Employment contracts were the predominant employment type (80.8%), and departmental distribution was relatively even, with medical and surgical nurses accounting for nearly half the total. Nurses with 11–15 years of experience were the most common regarding working conditions. Most participants (74.9%) worked an average of 7–8 h daily, while 47.6% earned less than 5,000 RMB monthly. Night shift workload was considerable, with 38.2% working ≥5 night shifts per month. Furthermore, 85.3% reported no recent major life events. This study found significant differences (*P* < 0.05) in turnover intention scores among the 798 clinical nurses based on nine demographic characteristics: age, marital status, professional title, type of employment, length of service in nursing, average daily working hours, monthly income, number of night shifts per month, and recent major life events.

**Table 1:** Participants' demographic characteristics and their distribution by turnover intention scores (*n* = 798).

**Characteristics**	***N* (%)**	**Turnover intention ($\bar{X}\pm S$)**	***t/F***	***P-*value**
**Gender**				
Male	31 (3.9)	13.06 ± 4.35	0.021	0.984
Female	767 (96.1)	13.05 ± 3.94		
**Age (years)**				
≤ 25	115 (14.4)	13.33 ± 4.19	9.917	< 0.001
26–30	207 (25.9)	13.42 ± 3.89		
31–40	322 (40.4)	13.37 ± 3.81		
41–50	124 (15.5)	11.99 ± 4.02		
≥51	30 (3.8)	10.33 ± 2.86		
**Marital status**				
Unmarried	195 (24.4)	13.59 ± 4.07	5.078	0.006
Married	573 (71.8)	12.96 ± 3.91		
Divorced/Widowed	30 (3.8)	11.27 ± 3.38		
**Having children or not**				
No	250 (31.3)	13.36 ± 4.01	1.479	0.140
Yes	548 (68.7)	12.91 ± 3.92		
**Highest educational attainment**				
Secondary and below	5 (0.6)	14.00 ± 3.81	0.518	0.670
Associate degree	200 (25.1)	12.85 ± 3.95		
Bachelor's degree	584 (73.2)	13.13 ± 3.96		
Master's degree and above	9 (1.1)	12.11 ± 3.86		
**Professional title**				
Nurse	147 (18.4)	13.29 ± 4.10	11.183	< 0.001
Nurse practitioner	261 (32.7)	13.12 ± 3.84		
Nurse practitioner in charge	326 (40.9)	13.18 ± 4.05		
Associate nurse practitioner	58 (7.3)	11.76 ± 3.29		
Director of nursing	6 (0.8)	9.50 ± 1.38		
**Job position**				
Clinical nurse	624 (78.2)	13.10 ± 4.03	0.159	0.959
Staff nurse	95 (11.9)	12.96 ± 3.49		
Charge nurse	45 (5.6)	12.84 ± 3.64		
Unit nurse manager	29 (3.6)	12.62 ± 4.30		
Nursing supervisor	5 (0.6)	12.80 ± 3.56		
**Type of employment**				
Tenured employee	148 (18.5)	12.05 ± 3.64	17.323	< 0.001
Agency employee	5 (0.6)	17.60 ± 2.07		
Contractual employee	645 (80.8)	13.24 ± 3.98		
**Department**				
Internal medicine	214 (26.8)	13.11 ± 4.01	1.805	0.083
Surgery	152 (19.0)	13.15 ± 4.15		
Obstetrics and gynecology	55 (6.9)	14.22 ± 3.45		
Pediatrics	41 (5.1)	12.68 ± 3.13		
Emergency department	27 (3.4)	13.89 ± 3.66		
Operating room	35 (4.4)	11.57 ± 3.70		
ICU	38 (4.8)	13.32 ± 4.01		
Other	236 (29.6)	12.80 ± 4.00		
**Length of service in nursing (years)**				
≤ 5	177 (22.2)	13.17 ± 4.10	6.973	< 0.001
6–10	200 (25.1)	13.64 ± 3.72		
11–15	219 (27.4)	13.38 ± 3.92		
>15	202 (25.3)	12.00 ± 3.90		
**Average daily working hours (hours)**				
< 7	7 (0.9)	13.00 ± 2.89	13.513	< 0.001
7–8	598 (74.9)	12.64 ± 3.86		
>8	193 (24.2)	14.32 ± 3.99		
**Monthly income (yuan)**				
< 5,000	380 (47.6)	13.46 ± 4.02	3.676	0.014
5,000–8,000	307 (38.5)	12.70 ± 3.87		
8,001–10,000	77 (9.6)	12.90 ± 4.09		
>10,000	34 (4.3)	11.91 ± 3.08		
**Number of night shifts per month**				
≤ 1	295 (37.0)	12.23 ± 3.78	11.339	< 0.001
2–4	198 (24.8)	13.21 ± 3.67		
≥5	305 (38.2)	13.74 ± 4.15		
**Recent major life events**				
No	681 (85.3)	12.85 ± 3.97	−3.525	< 0.001
Yes	117 (14.7)	14.23 ± 3.61		

### 3.3 Scores of the measurement scales

The scores for the main variables in this study were as follows: the mean score for nurses' turnover intention was (13.05 ± 3.95), indicating that the participants had some degree of turnover intention; the mean score for psychological violence in the workplace was (21.11 ± 26.87); and the mean score for perceived stress was (22.78 ± 7.79). The scores for each dimension of the Turnover Intention, Workplace Psychological Violence, and Perceived Stress scales are presented in Table 2.

**Table 2:** Scores of nurses' turnover intention, workplace psychological violence and perceived stress (*n* = 798).

**Scale**	**Dimension**	**Items**	**Total score ($\bar{X}\pm S$)**	**Average score ($\bar{X}\pm S$)**
Turnover intention		6	13.05 ± 3.95	2.18 ± 0.66
	Possibility of quitting	2	3.88 ± 1.71	1.94 ± 0.85
	Motivation to find other jobs	2	3.77 ± 1.71	1.88 ± 0.85
	Possibility of obtaining other jobs	2	5.40 ± 1.50	2.70 ± 0.75
Workplace psychological violence		32	21.11 ± 26.87	0.66 ± 0.84
	Individual isolation work	10	7.76 ± 9.40	0.78 ± 0.94
	Attack on professional status	9	7.42 ± 9.36	0.82 ± 1.04
	Attack on personality	7	3.63 ± 5.93	0.52 ± 0.85
	Direct negative behaviors	6	2.30 ± 4.47	0.38 ± 0.75
Perceived stress		14	22.78 ± 7.79	1.63 ± 0.56
	Sense of tension	7	12.25 ± 5.21	1.75 ± 0.74
	Sense of losing control	7	10.53 ± 6.06	1.50 ± 0.87

### 3.4 Study variable correlations

Table 3 presents the correlation analysis results for the dimensions of turnover intention, workplace psychological violence, and perceived stress. The Pearson correlation analysis showed a positive correlation between turnover intention and workplace psychological violence (*r* = 0.364, *P* < 0.01). There were also significant positive correlations between turnover intention and perceived stress (*r* = 0.423, *P* < 0.01) and between workplace psychological violence and perceived stress (*r* = 0.486, *P* < 0.01).

**Table 3:** Correlations analysis of turnover intention, workplace psychological violence, perceived stress (*n* = 798).

**Variables**	**TI**	**TI1**	**TI2**	**TI3**	**WPV**	**WPV1**	**WPV2**	**WPV3**	**WPV4**	**PS**	**PS1**	**PS2**
TI	1.000											
TI1	0.905^**^	1.000										
TI2	0.880^**^	0.836^**^	1.000									
TI3	0.603^**^	0.292^**^	0.227^**^	1.000								
WPV	0.364^**^	0.403^**^	0.394^**^	0.051	1.000							
WPV1	0.313^**^	0.352^**^	0.350^**^	0.025	0.936^**^	1.000						
WPV2	0.382^**^	0.417^**^	0.403^**^	0.072^*^	0.938^**^	0.841^**^	1.000					
WPV3	0.317^**^	0.349^**^	0.345^**^	0.045	0.901^**^	0.757^**^	0.777^**^	1.000				
WPV4	0.308^**^	0.344^**^	0.328^**^	0.046	0.883^**^	0.757^**^	0.745^**^	0.870^**^	1.000			
PS	0.423^**^	0.511^**^	0.486^**^	−0.021	0.486^**^	0.449^**^	0.500^**^	0.407^**^	0.388^**^	1.000		
PS1	0.394^**^	0.397^**^	0.412^**^	0.118^**^	0.409^**^	0.357^**^	0.441^**^	0.355^**^	0.315^**^	0.630^**^	1.000	
PS2	0.205^**^	0.316^**^	0.272^**^	−0.129^**^	0.273^**^	0.271^**^	0.264^**^	0.218^**^	0.227^**^	0.744^**^	−0.050	1.000

^**^P < 0.01, TI = Turnover intention total score; TI1 = Possibility of quitting (dimension); TI2 = Motivation to find other jobs (dimension); TI3 = Possibility of obtaining other jobs (dimension); WPV = Workplace psychological violence total score; WPV1 = Individual isolation work (dimension); WPV2 = Attack on professional status (dimension); WPV3 = Attack on personality (dimension); WPV4 = Direct negative behaviors (dimension); PS = Perceived stress total score; PS1 = Sense of tension (dimension); PS2 = Sense of losing control (dimension).

### 3.5 The mediating effect of perceived stress between psychological violence in the workplace and turnover intention

**Figure 1:**
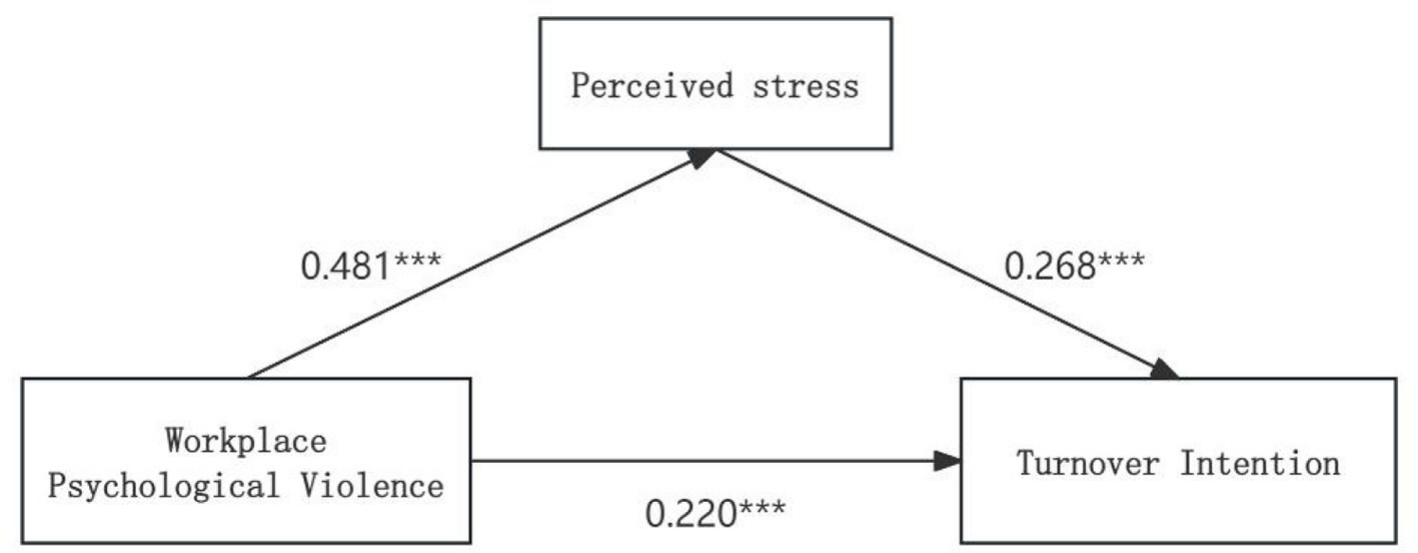
Mediation effect analysis of nurses' perceived stress between workplace psychological violence and turnover intention. ***P < 0.001.

The present study constructed a mediation effect model with turnover intention as the dependent variable, taking workplace psychological violence as the independent variable, perceived stress as the mediating variable, and while controlling for nine statistically significant variables derived from general demographic information. Table 4 presents the results of the mediation effect analysis using 5,000 bootstrap samples. The findings indicated that both workplace psychological violence and perceived stress were important predictors of turnover intention (β = 0.349, *P* < 0.001; β = 0.268, *P* < 0.001). Notably, even in the model incorporating perceived stress, workplace psychological violence still exerted a significant direct effect on turnover intention (β = 0.220, *P* < 0.001). Additionally, the present study identified a significant association between workplace psychological violence and perceived stress (β = 0.481, *P* < 0.001). Table 5 presents that the indirect effect was 0.129, with a 95% confidence interval (CI) of [0.094, 0.167]—a range that did not include zero, confirming the significance of the indirect effect. Specifically, perceived stress functioned as a significant mediator in this model, and the mediating effect accounted for 36.96% of the total effect. The relationships among these variables are illustrated in Figure 1.

**Table 4:** The mediating model of perceived stress between workplace psychological violence and turnover intention (*n* = 798).

**Outcome variable**	**Predictor variable**	***R***	***R* ^2^**	***F* (df)**	**β**	***t***
Turnover intention	Workplace psychological violence	0.458	0.210	20.930	0.349	10.702^***^
	Age				−0.133	−1.886
	Marital status				−0.119	−1.361
	Professional title				0.158	2.419^*^
	Type of employment				0.111	2.346^*^
	Length of service in nursing				−0.011	−0.175
	Average daily working hours				0.308	4.172^***^
	Monthly income				−0.117	−2.529^*^
	Number of night shifts per month				0.107	2.583^*^
	Recent major life events				0.224	2.459^*^
Perceived stress	Workplace psychological violence	0.547	0.299	33.506	0.481	15.659^***^
	Age				−0.183	−2.767^**^
	Marital status				0.110	1.331
	Professional title				−0.008	−0.135
	Type of employment				0.058	1.293
	Length of service in nursing				−0.019	−0.321
	Average daily working hours				0.198	2.843^**^
	Monthly income				0.023	0.524
	Number of night shifts per month				0.049	1.247
	Recent major life events				0.283	3.299^**^
Turnover intention	Workplace psychological violence	0.510	0.260	25.144	0.220	6.090^***^
	Perceived stress				0.268	7.305^***^
	Age				−0.084	−1.221
	Marital status				−0.149	−1.750
	Professional title				0.161	2.533^*^
	Type of employment				0.095	2.084^*^
	Length of service in nursing				−0.006	−0.098
	Average daily working hours				0.255	3.550^***^
	Monthly income				−0.123	−2.748^**^
	Number of night shifts per month				0.094	2.340^*^
	Recent major life events				0.148	1.669

^***^P < 0.001, ^**^P < 0.01, ^*^P < 0.05.

**Table 5:** Decomposition table of total effect, direct effect, and mediating effect (*n* = 798).

**Items**	**Effect**	**Boot SE**	**95% Boot LLCI**	**95% Boot ULCI**	**The relative effect (%)**
Total effect	0.349^***^	0.033	0.285	0.413	
Direct effect	0.220^***^	0.036	0.149	0.291	63.04%
Mediating effect	0.129^***^	0.019	0.094	0.167	36.96%

^***^P < 0.001.

## 4 Discussion

### 4.1 Current status of nurses' turnover intention

The global nursing shortage has reached critical levels, especially given the growing aging population, and retention of experienced nurses is essential to maintain continuity of care, patient safety, and healthcare system efficiency (37). Rural hospitals in China face critical healthcare staff shortages and high turnover rates (38). In this study, the nurses' turnover intention score was 13.05 ± 3.95, and 43.86% of the nurses had high turnover intention, which is consistent with findings by Huang et al. (28). This result mainly stems from the high-intensity and high-pressure characteristics of nursing work, as well as problems such as workload overload, understaffing, frequent overtime, and demanding shift work schedules, which seriously affect nurses' work-life balance and lead to physical and mental exhaustion and burnout (17, 39). Early attention to and intervention for nurses' turnover intention can help reduce nursing attrition and maintain nursing team stability. Nursing managers can demonstrate humanistic care through holiday support, reasonably arrange work schedules to prevent excessive fatigue, and provide relaxation spaces by establishing stress relief areas in departments to create a mutually supportive work environment.

### 4.2 Correlation analysis of psychological violence in the workplace, turnover intention, and perceived stress

The study showed a significant positive correlation between turnover intention and psychological violence in the workplace (*r* = 0.364, *P* < 0.001), which is consistent with findings by Luo et al. (11). Exposure to workplace psychological violence significantly predicted higher turnover intention (40). Psychological violence contributes to turnover intention by negatively affecting nurses' psychological and emotional wellbeing, leading to psychological problems such as low mood, anxiety, and depression (41, 42). Additionally, clinical nurses work in consistently high-pressure environments, and workplace psychological violence serves as an additional stressor that further increases nurses' psychological burden. This leads them to question their occupational value, reduces their professional identity and job satisfaction, and ultimately promotes turnover intention (43). Turnover intention was positively correlated with perceived stress (*r* = 0.423, *P* < 0.001), similar to findings by An et al. (44). Research shows that stress not only significantly affects employees' job satisfaction and organizational commitment, but is also a key predictor of turnover (45). According to stress coping theory (46), physiological and psychological strain responses occur when individuals face stressors that exceed their coping abilities, and turnover intention represents a critical manifestation of such strain responses. Workplace psychological violence was also positively correlated with perceived stress (*r* = 0.486, *P* < 0.001), suggesting that higher levels of workplace psychological violence experienced by nurses correspond to higher perceived stress levels. As an essential occupational stressor, workplace psychological violence significantly increases nurses' perceived stress by depleting their psychological resources and triggering psychological and physiological reactions such as anxiety, depression, and sleep disorders (47).

### 4.3 The mediating effect of perceived stress between psychological violence in the workplace and turnover intention

The results of the mediation analysis showed that nurses' perceived stress partially mediated the relationship between psychological violence in the workplace and turnover intention, accounting for 36.96% of the total effect. According to the Job Demands-Resources Model (JD-R) (22), psychological violence in the workplace, as a high-intensity job demand, depletes nurses' psychological resources. When job demands exceed an individual's existing resources, it activates the health impairment process within the model, inducing psychological strain in nurses and subsequently elevating perceived stress levels. Research findings confirm that workplace psychological violence not only directly induces nurses' intention to leave but also indirectly fosters this intention by elevating their perceived stress levels. Specifically, workplace psychological violence threatens nurses' psychological safety, undermines their trust in the work environment, significantly increases perceived stress levels, intensifies work-related pressure, and negatively impacts nurses' work attitudes and professional commitment, ultimately resulting in additional organizational losses (17, 48). Therefore, reducing nurses' perceived stress by minimizing psychological violence in the workplace is an effective way to prevent and reduce nurses' turnover intention. When healthcare organizations develop interventions, they should not only reduce violence at its source but also focus on alleviating nurses' perceived stress. Nursing managers should introduce clear anti-psychological violence policies, improve anonymous reporting mechanisms (such as dedicated complaint systems with specialized personnel), and establish standardized processing procedures. They should foster a zero-tolerance culture toward psychological violence and create a supportive work atmosphere through regular anti-violence seminars, effective communication mechanisms, and educational programs. Simultaneously, efforts should focus on alleviating nurses' perceived stress by establishing emotional support channels, providing anonymous psychological support platforms, creating VR-based relaxation environments, and offering professional psychological counseling services to help nurses address negative emotions and trauma from psychological violence. Organizations can improve nurses' job satisfaction, reduce turnover intention, and maintain nursing team stability by preventing psychological violence and relieving perceived stress.

## 5 Limitations

This study employed convenience sampling, with the sample drawn exclusively from nursing populations at nine Grade III Class A general hospitals in Southwest China. This approach may introduce sample bias and limit representativeness. Additionally, as a cross-sectional survey design, the study can only reveal associations among workplace psychological violence, perceived stress, and turnover intention among nurses. The causal relationships among these three factors require longitudinal research to confirm. Furthermore, all variables were measured through self-reporting, which may introduce bias. For instance, nurses might withhold truthful responses regarding psychological violence experiences or turnover intention due to concerns. Future research should include nursing populations from other regions of China and employ random sampling to enhance sample representativeness and generalizability. Concurrently, qualitative studies and longitudinal investigations should be actively pursued.

## 6 Conclusions

This study provides insight into the mediating role of perceived stress among clinical nurses in the relationship between workplace psychological violence and turnover intention. The findings suggest that workplace psychological violence significantly increases nurses' perceived stress levels, which in turn increases their turnover intention, and that perceived stress plays a key mediating role in this process. This finding reveals the importance of alleviating nurses' perceived stress in mitigating the adverse consequences of workplace psychological violence, and provides direction for developing targeted intervention strategies. By creating a violence-free work environment, conducting stress management training, and providing psychological support services, organizations can effectively prevent psychological violence and reduce nurses' perceived stress, thereby decreasing turnover intention. By integrating intervention measures with nurse protection systems, workplace psychological violence can be effectively prevented, nurses' professional identity can be enhanced, and nursing team stability can be maintained.

## Data Availability

The raw data supporting the conclusions of this article will be made available by the authors, without undue reservation.
